# Patient and health professional views on risk-stratified monitoring of immune-suppressing treatment in adults with inflammatory diseases

**DOI:** 10.1093/rheumatology/keae175

**Published:** 2024-03-14

**Authors:** Amy Fuller, Jennie Hancox, Hywel C Williams, Tim Card, Maarten W Taal, Guruprasad P Aithal, Christopher P Fox, Christian D Mallen, James R Maxwell, Sarah Bingham, Kavita Vedhara, Abhishek Abhishek

**Affiliations:** Academic Rheumatology, School of Medicine, University of Nottingham, Nottingham, UK; School of Health Sciences, Loughborough University, Loughborough, UK; Lifespan and Population Health, University of Nottingham, Nottingham, UK; Lifespan and Population Health, University of Nottingham, Nottingham, UK; Lifespan and Population Health, University of Nottingham, Nottingham, UK; Centre for Kidney Research and Innovation, Translational Medical Sciences, University of Nottingham, Derby, UK; Nottingham Digestive Diseases Centre, Translational Medical Sciences, University of Nottingham, Nottingham, UK; NIHR Nottingham Biomedical Research Centre (BRC), Nottingham University Hospitals NHS Trust and The University of Nottingham, Nottingham, UK; School of Medicine, University of Nottingham, Nottingham, UK; Primary Care Centre Versus Arthritis, Keele University, Keele, UK; Department of Rheumatology, Sheffield Teaching Hospitals NHS Foundation Trust, Sheffield, UK; Department of Rheumatology, Norfolk and Norwich University Hospitals NHS Foundation Trust, Norwich, UK; Lifespan and Population Health, University of Nottingham, Nottingham, UK; School of Psychology, Cardiff University, Cardiff, UK; Academic Rheumatology, School of Medicine, University of Nottingham, Nottingham, UK; NIHR Nottingham Biomedical Research Centre (BRC), Nottingham University Hospitals NHS Trust and The University of Nottingham, Nottingham, UK

**Keywords:** immune-mediated inflammatory disease, steroid sparing drugs, blood monitoring, qualitative

## Abstract

**Objective:**

To explore the acceptability of an individualized risk-stratified approach to monitoring for target-organ toxicity in adult patients with immune-mediated inflammatory diseases established on immune-suppressing treatment(s).

**Methods:**

Adults (≥18 years) taking immune-suppressing treatment(s) for at least six months, and healthcare professionals (HCPs) with experience of either prescribing and/or monitoring immune-suppressing drugs were invited to participate in a single, remote, one-to-one, semi-structured interview. Interviews were conducted by a trained qualitative researcher and explored their views and experiences of current monitoring and acceptability of a proposed risk-stratified monitoring plan. Interviews were transcribed verbatim and inductively analysed using thematic analysis in NVivo.

**Results:**

Eighteen patients and 13 HCPs were interviewed. While participants found monitoring of immune-suppressing drugs with frequent blood-tests reassuring, the current frequency of these was considered burdensome by patients and HCPs alike, and to be a superfluous use of healthcare resources. Given abnormalities rarely arose during long-term treatment, most felt that monitoring blood-tests were not needed as often. Patients and HCPs found it acceptable to increase the interval between monitoring blood-tests from three-monthly to six-monthly or annually depending on the patients’ risk profiles. Conditions of accepting such a change included: allowing for clinician and patient autonomy in determining individuals’ frequency of monitoring blood-tests, the flexibility to change monitoring frequency if someone’s risk profile changed, and endorsement from specialist societies and healthcare providers such as the National Health Service.

**Conclusion:**

A risk-stratified approach to monitoring was acceptable to patients and health care professionals. Guideline groups should consider these findings when recommending blood-test monitoring intervals.

Rheumatology key messages

- Risk-stratifying monitoring blood-tests during established immune-suppressing drug treatment is cost-effective, but its acceptability is unknown.
- Patients and health professionals found it acceptable to extend monitoring blood-test intervals based on individualized risk profiles.
- Monitoring guidelines could change, reducing the burden of monitoring on patients’ and healthcare systems.

## Introduction

Immune-mediated inflammatory diseases (IMIDs) such as rheumatoid arthritis (RA), inflammatory bowel disease (IBD), psoriasis (PsO) ± arthritis (PsA), ankylosing spondylitis (AS) and systematic lupus erythematosus (SLE) together affect over 4% of adults [1–6]. They are treated with long-term steroid sparing disease-modifying anti-rheumatic drugs (DMARDs), which can cause hepatotoxicity, myelotoxicity and nephrotoxicity. Those prescribed these medicines undergo regular blood tests to check for such side-effects, typically fortnightly-to-monthly when treatment is commenced and three-monthly once treatment becomes stable [7–10]. These side-effects seldom occur during stable long-term treatment [11–16]. The practice of undertaking three-monthly monitoring blood tests for all patients is based on expert opinion from guideline writing groups, often underpinned by the summary of product characteristics. Performing these tests at fixed intervals regardless of individuals’ risk is an unjustifiable use of resources and goes against the tenets of personalized medicine.

We have developed risk-stratified monitoring strategies for methotrexate, leflunomide, thiopurine, sulfasalazine and 5-aminosalicylate toxicity [12, 15–18]. These consider individuals’ risk of developing clinically significant side-effects to determine their individualized frequency of monitoring blood tests, rather than having a standard approach for all. A health economic analysis based upon these risk predictions revealed that this approach was more cost-effective than current practice [11, 12]. For anti-TNF-alpha (α) drugs, we evaluated the cost-effectiveness of different blood-test monitoring strategies to ascertain the most cost-effective strategy due to low outcome event rate and availability of a single dataset that precluded prognostic model development [11].

Before this new evidence is used to change guidelines and clinical practice, it is vital to explore whether such changes would be acceptable to patients and healthcare professionals (HCPs). Therefore, this study explored the views and experiences of people with IMIDs and HCPs managing their treatment, about current monitoring practice and the acceptability of a risk-stratified monitoring strategy.

## Methods

### Study design

This is a multicentre, qualitative interview study.

### Participants

#### Patients

Adults aged ≥18 years self-reporting physician-diagnosed RA, IBD, PsO and/or PsA, AS or SLE, and treated with conventional DMARDs or anti-TNF-αs for six months or longer comprised the patient participants. They were recruited from dermatology, gastroenterology and rheumatology clinics in National Health Service (NHS) hospitals or via advertisements promoted by patient organizations (see acknowledgements) in their online newsletters, webpages and social media platforms. Patients answered a questionnaire (Supplementary Data S1, available at *Rheumatology* online) to assess eligibility for interview and to recruit people representing the broad range of conditions, treatments and engagement with monitoring. A combination of purposive stratified and maximum variation sampling was employed to recruit participants with different IMIDs, treatments, risk factors for drug toxicity and levels of adherence to monitoring recommendations.

#### HCPs

HCP participants comprised doctors [consultants and general practitioners (GP)]; and allied health professionals (specialist nurses and pharmacists) with experience of prescribing and/or monitoring DMARDs. The latter group was included because specialist nurses and pharmacists prescribe immune-suppressing drugs and participate in their monitoring in the UK. HCPs were recruited using a snowballing technique [19] and through national associations’ mailing lists. Purposive sampling was employed to recruit a mix of HCPs working in rheumatology, dermatology, gastroenterology or primary care. They completed a brief questionnaire to assess their eligibility for interview.

### Data collection

Single, one-to-one, semi-structured interviews were conducted remotely by A.F., an experienced qualitative research fellow, who made the initial email contact and recruited participants. At the start of interviews, it was explained to participants that A.F. was not involved in patient care and would remain impartial to their views. Interviews were digitally audio-recorded and transcribed verbatim.

Separate interview guides were developed for patients and HCPs (Supplementary Data S2 and S3, available at *Rheumatology* online). Two Patient and Public Involvement (PPI) volunteers with IMIDs treated with immune-suppressing medications advised on the patient questionnaire, interview guide and interview format.

The interview guides were in two parts. Part 1 for patients explored their experience of current monitoring blood tests, reasons for adherence or non-adherence, perceived risks and benefits, and view on the importance of continuing with current monitoring. Part 1 for HCPs explored the practicalities and perceived risks and benefits of current monitoring.

In Part 2, for both patients and HCPs, the risk-stratified monitoring strategy was introduced. This covered the development and deployment of a risk calculator that resulted from the prior work and determined a person’s individual risk of developing side-effects from their IMID treatment, presented as a score.

With patient participants taking conventional DMARDs, A.F. computed and presented their risk score using the calculator and discussed the different potential frequencies of monitoring that the health economic analysis demonstrated would be cost-effective: six-monthly, annually and biennially. Patient participants taking anti-TNF-αs were informed of the overall rate of side-effects and presented with the potential frequencies of monitoring.

HCPs were presented with four-to-five anonymized descriptive scenarios representing a range of risk profiles (Supplementary Data S4, available at *Rheumatology* online). Participant acceptability, concerns and perceived risks and benefits of changing to the different frequencies of monitoring blood tests were then explored.

### Risk calculator and score

The risk calculator, developed in prior work [12, 15–18], considers different prognostic factors to give an overall risk score. The risk score is expressed as the percentage of people with the same characteristics that would have to stop treatment due to an abnormal blood-test result over 12 months.

Although broadly similar, the exact prognostic factors and how much they influence the risk score are unique to each DMARD [12, 15–18].

### Data analysis

Anonymized transcripts were analysed thematically using an inductive approach [20]. Analysis was managed using NVivo (v12), taking place in parallel with data collection so initial results informed subsequent sampling and data collection. Analysis of the first four patient and six HCP interviews was performed independently by A.F. and J.H., noting initial meanings, patterns and codes. They came together repeatedly to discuss and generate an initial coding framework of data-driven themes and identify areas for further exploration. With good agreement in coding, A.F. analysed the remaining interviews and further developed the coding framework using the principles of constant comparison to refine and ensure preliminary themes were consistent with the rest of the interviews. A.F., J.H. and A.A. (rheumatology and medicine expertise) also came together to discuss the preliminary themes and clarify clinical concepts that supported coding and theme development. Following analysis of the 18th patient and 13th HCP interview, no further changes were made to the coding framework. Thus, it was concluded sufficient saturation of the data had been achieved and data collection ended.

### Ethical approval

West Midlands-Black Country Research Ethics Committee (Ref: 21/WM/0285). Participants gave their informed consent via an online consent form prior to the interview.

## Results

Eighteen patient and 13 HCPs were interviewed (Table 1). Patients were predominantly female, white ethnicity and had a range of IMIDs and treatments. Their risk scores ranged between 1% and 3%. The HCPs included consultants, GPs, nurses and pharmacists from four specialisms. Patient and HCP interviews lasted for 50 (range 38–57) and 44 (range 20–61) min on average, respectively. Four themes with eight subthemes were generated in the data. These are presented in Table 2 with accompanying illustrative quotes, which are denoted in the text with a letter.

**Table 1.** Study participants

Patients	*n* = 18
Age (years), range	21–67
Female gender, *n* (%)	13 (72)
White ethnicity, *n* (%)	18 (100)
Diagnosis, *n* (%)	
Rheumatoid arthritis	3 (17)
Ankylosing spondylitis	3 (17)
Systemic lupus erythematosus	2 (11)
Ulcerative colitis	2 (11)
Crohn’s disease	4 (22)
Skin psoriasis	4 (22)
Adherence to monitoring, *n* (%)	
Good (>80% attendance)	12 (67)
Poor (<80% attendance)	6 (33)
Current immunosuppressant, *n* (%)^a^	
Methotrexate	7 (39)
Sulfasalazine	1 (6)
Leflunomide	1 (6)
Azathioprine	3 (17)
Mercaptopurine	1 (6)
Anti-TNF-alpha monotherapy	5 (28)
Duration on current immunosuppressant, *n* (%)	
1–2 years	3 (17)
2–3 years	3 (17)
3–4 years	5 (28)
5–10 years	4 (22)
>10 years	3 (17)
Recommended monitoring frequency, *n* (%)	
Fortnightly	1 (6)
Monthly or bi-monthly	3 (17)
Three-monthly	13 (72)
Six-monthly	1 (6)
Predicted risk (%) of stopping treatment due to abnormal blood test result in the next 12 months, *n* (%)^b^	
1%	5 (28)
2%	6 (33)
3%	2 (11)

Health professionals	*n* = 13

Job role, *n* (%)	
Consultant	6 (46)
Specialist nurse or pharmacist	4 (31)
General practitioner	3 (23)
Speciality, *n* (%)	
Rheumatology	5 (38)
Dermatology	3 (23)
Gastroenterology	2 (15)
Primary care	3 (23)
Female, *n* (%)	7 (54)
Years in speciality	
<5 years	1
5–10 years	2
10–20 years	5
>20 years	5

a Three participants were taking a DMARD and anti-TNF-alpha (combined therapy).

b No risk-score for anti-TNF-alpha monotherapy.

**Table 2.** Themes, subthemes and supporting quotes

Theme: Benefits and challenges to current monitoring
Subtheme 1: Reassurance and continuity of care a. It’s more the peace of mind and just knowing that everything is okay. *Patient 5, RA* b. I saw it as the medication was what was going to help me, and if one of the requirements was to have regular blood tests then so be it. *Patient 17, AS* c. It does give me that reassurance that you’re still part of that system, that someone’s still looking out for you. *Patient 12, IBD*
Subtheme 2: Incidental findings leading to the diagnosis of another condition d. Incidental findings that the physicians are not aware of and then that allows early interventions … but they might be few and far between. *HCP 2, dermatologist*
Subtheme 3: Practical challenges of frequent monitoring e. When it’s time to get my prescription every third month, there’s always a hiccup, it’s always late and I always have to chase it. So, I’m at the stage of running out or not having any [medication] to take, because the results aren’t filtering through. *Patient 13, PsO* f. The powers that be just don’t consider how many people are involved in the stage of getting the drug to the person and how much time it takes for each person to do that. *HCP 12, GP*
Subtheme 4: Remembering to book a test and consequences of non-compliance g. In my case, you’ve got other things wrong with you as well. And depending on how severe they are at the time I can sort of prioritize those, so you then suddenly forget about the need to do your blood test. *Patient 15, AS* h. Patients will often flare in terms of their disease, so that causes issues that could have been prevented if they continued on their regimen. *HCP 10, rheumatology pharmacist*
Subtheme 5: Clinicians’ interpretation and actioning of monitoring results i. We don’t have any consistent guidelines on what to action and what not to action, so then I feel like a lot of the time we’re probably doing unnecessary bloods … every consultant will do things differently here. *HCP 6, dermatology nurse*
Theme: Questioning the need for the current frequency of monitoring
j. Because I’ve been on azathioprine for a few years now, you know, I seem to have settled with it. I think a six-monthly blood test would be better. *Patient 8, IBD* k. I can’t remember the last time I saw an abnormal [result] … we’re just taking tonnes of bloods, and nothing happens. *HCP 13, GP* l. During COVID time … our patients could not go in … so that prompted you to say, ‘Do we need to have blood monitoring that frequently?’ … for the last two years, how many bad side effects or problems you were faced with? That is negligible. *HCP 3, rheumatologist*
Theme: Adopting a risk-stratified monitoring plan
Subtheme 1: Views on risk scores and acceptability of proposed frequencies m. One out of 100, that doesn’t seem like a large amount. That gives me a bit of reassurance. *Patient 1, PsO (risk score 1);* So, I’m quite low risk then really. *Patient 3, IBD (risk score 3)* n. I think it’s too big a jump and I think if there were any issues, one year since the last test, who knows what could have happened. Whereas if you have the test sooner, things are picked up and can be treated. *Patient 3, IBD* o. We treat lots of patients with anti-TNF drugs. Although they have lots of other issues, we seldom stop it because of repeat blood test abnormalities. I would be happy with once a year on that basis. *HCP 8, gastroenterologist* p. You could do it to six months and do that for three years or two years and then move to a year if it seems safe and appropriate. *HCP 11, GP* q. I suppose if you just questioned the 1 out of 100, then I’d be more inclined to say okay six monthly or once a year is fine, less frequent. But if you gave me the specific context of the medication, then I would change my view. Only because I personally don’t think from anecdotal clinical practice that it is 1 out of 100 in this situation, it’s probably more. *HCP 9, gastroenterologist*
Subtheme 2: Provisos to accepting a reduced monitoring schedule r. I think six months with good feedback is acceptable absolutely, I would be happy with that if I was, as long as I could get the results and I know what was going on, I’d be very happy with six months. *Patient 6, RA* s. This needs to be consistent across every indication for this drug, rheumatological, dermatological, hepatological, whatever it is, it needs to be the same. *HCP 8, gastroenterologist*
Subtheme 3: Perceived impact of proposed strategy t. The less that psoriasis can get in the way of my everyday life, the better, and that includes like hospital appointments and blood tests. *Patient 4, PsO* u. Where consultants will prescribe, one of the biggest factors will be that they can use that time to actually see more of the patients who are being referred to them. So those patients get treated faster. *HCP 7, rheumatology pharmacist*
Theme: Communicating a change in practice
v. There’s a lot of difficulty letting go of the old ways … You might need the odd champion to go round and speak to people in person. *HCP 4, dermatologist* w. Definitely like a good leaflet or a handout that we could send to the patients would be really good, explaining the reasons why we’ve decided to change the frequency. *HCP 6, dermatology nurse*

Patient participant quotes identified by participant number and inflammatory condition; HCP participant quotes identified by participant number, job role and specialism.

AS: ankylosing spondylitis; IBD: inflammatory bowel disease; PsO: skin psoriasis; RA: rheumatoid arthritis.

### Benefits and challenges to current monitoring

#### Reassurance and continuity of care

Both patients and HCPs found regular monitoring reassuring, to know that the treatment was not causing side-effects and could be stopped early should abnormal results arise (a). This was a key reason for patients adhering to monitoring blood tests. Conversely, some patients viewed monitoring as a tick-box exercise to continue receiving their prescription (b). Patients and HCPs alike said the regularity of monitoring provided a feeling of continuity of care through regular contact between patients and prescribers (c).

#### Incidental findings leading to the diagnosis of another condition

HCPs said that frequent blood tests meant other conditions including comorbidities were occasionally detected early and could be investigated or treated promptly; however, they acknowledged this was uncommon (d).

#### Practical challenges of frequent monitoring

Organising and attending their monitoring blood tests was time consuming and inconvenient for many patients because of work commitments, difficulties securing an appointment, or having to chase results to receive their prescription (e). HCPs noted that monitoring was resource-intensive, taking up a large proportion of their workload. Capacity issues were highlighted within busy NHS settings to provide and review tests at the current frequency (f).

#### Remembering to book a test and consequences of non-compliance

While many participants ensured they had timely blood tests so that their prescription would be renewed, others often forgot to do so because they lost track of time, were busy, prioritized other health issues or didn’t receive a prompt before the test was due. These were common reasons for non-compliance (g). HCPs reported that when patients missed a blood test, they were unable to issue a prescription, risking the patients’ flaring or having a poorly controlled IMID (h).

#### Clinicians’ interpretation and actioning of monitoring results

HCPs discussed how abnormal test results were often false positives or transient abnormalities not caused by treatment, such as raised liver markers due to excessive alcohol consumption before a blood test, but still required investigation or repeat testing. This resulted in unnecessary concern for patients, and risked an IMID flaring if medication was paused.

Some HCPs highlighted how clinicians had different thresholds for investigating abnormalities, with the perception that some blood tests are repeated unnecessarily (i).

### Questioning the need for the current frequency of monitoring

Most patients questioned why they should continue with such frequent monitoring given their IMID, medication dosage and test results had remained stable, and felt it could be reduced (j). Many HCPs viewed the current approach to monitoring as outdated and overly cautious. Based on their clinical experience, abnormalities arising from DMARDs were uncommon once a patient was established on a stable dose, and thus a reduction in frequency would be plausible (k). Some HCPs were already working to reduced monitoring schedules for medications considered to be a low risk of causing side effects, such as anti-TNF-αs and sulfasalazine. A few also reported that there were no observable increases in the rate of abnormal results during the COVID-19 pandemic lockdown when they had to implement reduced monitoring schedules. This made them question the need to return to three-monthly monitoring (l).

### Adopting a risk-stratified monitoring plan

#### Views on risk scores and acceptability of proposed frequencies

Most patients perceived their risk of stopping treatment to be low. A small number of patients with scores of 1% and 2% per year interpreted their risk as a little high, but despite this had similar views on the potential monitoring frequencies as other patients (m). All patients felt six-monthly testing was a suitable monitoring frequency, either as a comfortable stepdown or reflective of how often they currently had a blood test. Some were happy to reduce to annual monitoring and felt it could tie in with their annual consultations, although several stipulated monitoring should be tapered down rather than suddenly move to yearly. A few patients were uncomfortable with annual monitoring, considering it too long for side effects to be left undetected (n).

HCPs were in favour of adopting a risk-stratified approach to monitoring blood tests. All were happy for anti-TNF-αs to be monitored annually, and viewed as rarely or never causing the blood-test abnormalities that can arise from conventional DMARDs (o).

For conventional DMARD scenarios, HCPs viewed risk scores of 1–2% per year as low risk with acceptable frequencies being either six-monthly or annually. Risk scores of 3% or 4% per year were generally viewed by HCPs as higher risk, acceptable frequencies included staying at three-monthly, six-monthly or annually. Several also suggested tapering as a reassuring approach if annual monitoring was implemented, enabling them to see results are stable at a slightly lower frequency (i.e., six-monthly testing), before further increasing the gap between blood-tests (p). Most patients and HCPs were uncomfortable with biennial monitoring given the potential toxicity of the medications. While some HCPs used the risk scores to determine which monitoring frequency they found acceptable, several factored in their clinical experience and opinion of individual risk factors presented in the scenarios, which sometimes changed their viewpoint (q). GPs were less resolute than the other HCPs about the frequency of monitoring, and were happy to follow whatever was recommended by national guidance.

#### Provisos to accepting a reduced monitoring schedule

All participants had conditions or requests related to accepting a reduction in monitoring frequency.

While most patients welcomed NHS cost savings, many stressed that they would expect assurance this would not come at a cost to their care and safety. Some also wanted to receive feedback on their results, rather than having to assume everything was stable as they did currently (r).

For HCPs, consensus and endorsement from national level organisations and clinical specialty bodies was deemed necessary before any change in practice would be implemented (s). Some HCPs wanted guidance on how to interpret the risk score and select a suitable frequency, but also flexibility for them to override a recommended frequency (e.g., for patients considered particularly high-risk), and to accommodate patients’ preferences. Being involved in the decision was also important to some patients. Both participant groups felt there should be regular review of individuals’ risk score and the ability to change monitoring frequency where a person’s risk changed, although there were concerns that completing individual risk calculators would be a time-consuming exercise for clinicians.

#### Perceived impact of proposed strategy

Participants noted that reducing the frequency of monitoring blood tests would reduce the burden of IMIDs on patients’ lives, as they would spend less time organising, attending, and for some, anxiously awaiting the results of blood tests (t).

HCPs said not only would it reduce costs associated with providing monitoring of blood tests, it would also free up time to see patients earlier in their disease trajectory and provide more medication counselling and advice on managing their IMID (u).

While some HCPs felt reducing the frequency of monitoring may reassure patients about the safety of their medication, they too highlighted some may become more complacent or forgetful towards monitoring. Some patients said they would need to be prompted with the larger gap between blood tests.

### Communicating a change in practice

HCPs felt that any changes should be communicated through various channels (direct to clinicians, through specialist societies and/or articles in medical publications and magazines). Furthermore, they felt while a risk-stratified approach would be well received by most clinicians, some would need persuading beyond an update to national guidance (v). Additional detail about the research evidence behind the proposed changes was wanted by some HCP participants before they could say they would fully accept it, including an explanation of the prognostic factors included and excluded from the risk calculator and the weight each factor contributes towards the risk score. Given that they have been emphasizing the importance of three-monthly testing to their patients, HCPs felt that information leaflets and disseminating changes in practice through patient organizations would be necessary to support any verbal explanation they could provide (w).

## Discussion

This study explored patient and HCP views on monitoring for toxicity due to DMARDs using periodic blood tests during established treatment. It demonstrated the burden placed on patients and HCPs from such testing and their appetite to reduce it, with monitoring considered reassuring but the current recommended frequency unnecessary. A risk-stratified approach to increasing the interval between monitoring blood tests was acceptable although acceptability reduced with increasing gaps between tests. Adoption of this strategy into practice was dependent upon endorsement from specialist societies and healthcare organizations, and flexibility in being responsive to clinical need and patient preference, in keeping with principles of shared decision-making.

There is a need to manage the burden of treatment on patients with long-term conditions [21] and move away from a one-size-fits-all approach. Our study demonstrates that risk-stratified monitoring is acceptable to both patients and HCPs with potential for positive impacts to individuals and health systems by reducing the burden of monitoring, and minimizing pauses in continuous treatment due to missed blood tests and/or insignificant abnormalities. The views of different types of HCPs were similar.

For it to be adopted, a change in guidelines is required; this was an important condition of HCP acceptability. Given DMARDs are used across different specialities, efforts should be targeted towards changing the overarching monitoring recommendations, such as those issued by the National Institute of Health and Care Excellence, the Medicines and Healthcare products Regulatory Agency and manufacturing authorization holders.

The health economic exercise we previously carried out suggested that six-monthly, annual, or biennial monitoring frequencies would all be more cost-effective than three-monthly monitoring [11,12]. All patients were comfortable with reducing the frequency of their testing to every six months; however, some HCPs were hesitant to reduce monitoring beyond three-monthly for those with a risk score of ≥3% per year. It was unusual for patients to have such high risk; however, as patients occasionally do so, care must be taken when implementing risk-stratified monitoring [11–16]. With variability in accepted frequencies amongst HCPs, and the preference by some to have autonomy in their decision-making, monitoring recommendations may need to be given for risk score ranges. For example, six-monthly to annual monitoring for risk scores up to 2% per year, and three- to six-monthly for risk scores over 2% per year. All HCPs were happy to move to annual monitoring for anti-TNF-αs, suggesting a different recommendation could be made for such medications. The recommended monitoring frequency may change over time, e.g. as the patient accrues comorbidities. The most feasible way to implement risk-stratified monitoring is to integrate the calculator as an application in GP electronic health records. Such an application will automatically produce a risk score and recommend any changes in monitoring frequency each time a prescription is issued. Alternatively, this can be decided by the GP and/or the specialist during patients’ annual reviews.

Should annual monitoring be considered for national guidance, tapering may increase the likelihood it is accepted by patients and prescribers given many participants were more comfortable with this over moving straight to annual monitoring.

Some HCPs put more emphasis on individual prognostic factors such as age, lifestyle factors, other medications, and comorbidities rather than on the overall risk score. The acceptance of moving to annual monitoring for anti-TNF-αs appeared to be facilitated by this chiming with HCPs’ clinical experience of rarely seeing side-effects in those treated with these drugs. To minimize such preconceptions from impeding the interpretation of the risk score, it would be essential to communicate that HCPs should not focus on individual factors, but the total score given is based on the most recent evidence and takes all prognostic factors into account.

Furthermore, some HCPs wanted more information about how the risk-stratified approach was created to feel comfortable adopting it. To ensure HCP support and trust in adopting a risk-stratified approach to monitoring, a clear explanation of how the calculators producing risk scores were developed should also be included. This should also be considered when explaining changes to monitoring with patients given that participants had the risk score calculated with and explained to them, which may have provided important reassurance that facilitated their view that less frequent monitoring would be acceptable.

Strengths of this study include nationwide recruitment with findings reflecting the experiences of those receiving and providing medical care from different hospitals. There was a broad eligibility criterion for patients with different IMIDs, on different medications, and different HCP roles representing each disease area, enhancing transferability of the findings. Monitoring of long-term immune-suppressing treatments is done by GPs and a strategy that includes all conditions allows for ease of implementation across different conditions. Participants were informed that the interviewer was not involved in the care of patients to encourage honest sharing of opinion, minimize response bias and convey equipoise in whether a monitoring frequency reduction was acceptable or not. Data collection and analysis were conducted concurrently, which allowed for identification of areas requiring further exploration in the following interviews. With no changes to the codebook upon analysis of the final patient and HCP interviews, we can be confident that we reached a sufficient level of data saturation for the findings to be clinically meaningful and transferable [22]. Involvement of a second coder and clinical investigator enhanced the rigour of analysis. Our use of purposive sampling ensured a mix of patients with different levels of adherence to their recommended monitoring frequency were interviewed, and less adherent patients were forthcoming in discussing reasons for non-compliance.

Limitations include a lack of patient participants with an annual risk score >3% who may feel more cautious about adopting a reduced frequency of monitoring; however, most patients in the model development and validation populations had annual risk scores <3%. There were also no patient participants of non-white ethnicity, whose views and experiences may differ. We were unable to engage with them despite inviting many patients. Adherence to current monitoring may have been overestimated as it was self-reported. Burden from excessive monitoring blood tests is also an issue for paediatric patients but was not addressed in this study.

A risk-stratified approach to monitoring was acceptable to patients and HCPs. Recommendations towards adopting such a strategy in clinical practice should consider the preferred gaps in testing, tapering, clinician and patient autonomy in deciding appropriate monitoring frequencies, and flexibility to change monitoring frequency according to need.

## Supplementary material

Supplementary material is available at *Rheumatology* online.

## Supplementary Material

keae175_Supplementary_Data

## Data Availability

Deidentified data are available upon reasonable request to the corresponding author.
