# Supplementary material for: Patient and health professional views on risk-stratified monitoring of immune-suppressing treatment in adults with inflammatory diseases
Source: Rheumatology (Oxford). 2024 Mar 14;64(3):969–75. doi: 10.1093/rheumatology/keae175 (PMC11879332; doi:10.1093/rheumatology/keae175)

**Supplementary Data S1**

Patient eligibility questionnaire

| 1. Please tell us if you have been diagnosed with or suffered from any of the following medical problems?   *Tick all that apply. If none, please tick this box and move to question 2.* | Heart attack or angina / Heart failure / Other heart disease / High blood pressure (hypertension) / High levels of fats, lipids or cholesterol / Type 2 diabetes / Chronic kidney disease / Cancer, excluding skin cancer | |
| --- | --- | --- |
| 1. Please tell us if you have been diagnosed with any of the following conditions.   *Tick all that apply.* | Rheumatoid arthritis / Psoriatic arthritis / Inflammatory arthritis / Ulcerative colitis / Crohn’s disease / Skin psoriasis (without arthritis) / Ankylosing spondylitis / Lupus (SLE) / Other (please specify) | |
| 1. Place an X in the table below to tell us which of the following medications you are currently prescribed and whether you have taken these medicines for less than six months or longer than six months.   I*f you are not currently prescribed any of these medicines, please tick this box and move to question 7. Choose as many options as appropriate* | *I have been taking this medicine for less than six months*  Methotrexate  Azathioprine  Mercaptopurine  Mesalazine  Olsalazine  Sulfasalazine  Mycophenolate mofetil  Leflunomide  Biologic (anti-TNF-α) agents e.g. etanercept, adalimumab, infliximab, golimumab, or certolizumab  Other | *I have been taking this medicine for more than six months*  Methotrexate  Azathioprine  Mercaptopurine  Mesalazine  Olsalazine  Sulfasalazine  Mycophenolate mofetil  Leflunomide  Biologic (anti-TNF-α) agents e.g. etanercept, adalimumab, infliximab, golimumab, or certolizumab  Other |
| 1. How often are you advised to have a blood test for each of these medications?   *Please add the name of the medication(s) you are taking below and indicate with an X how often you are advised to have a blood test for each one. We have provided space for up to three different types of medication.* | [Medication name]  Fortnightly /  Monthly  Every two months  Every three months  Every four months  Every six months  Annually  No blood tests needed | |
| 1. Since starting this treatment, how often have you missed a monitoring blood test OR delayed attending a monitoring blood test by more than one month?   *This does not include any tests that were cancelled or delayed due to COVID-19.* | Never / About 1 out of every 10 appointments / About 2 out of every 10 appointments / About 3 out of every 10 appointments / About 4 or more of every 10 appointments | |
| 1. Where do you go to get your monitoring blood test? | GP practice / Local hospital / Other (please specify) | |
| 1. How old are you at present? | Free text | |
| 1. What is your gender? | Free text | |
| 1. What is your height? | Free text | |
| 1. What is your weight? | Free text | |
| 1. Which of the following best describes your ethnicity? | White / Mixed/multiple ethnic groups / Asian or Asian British / Black/African/Caribbean/Black British / Other ethnic group (please specify) | |
| 1. Do you have any educational qualifications for which you received a certificate? | Yes / No | |
| 1. If no, do you have any professional, vocational or other work-related qualifications for which you received a certificate? | Yes / No | |
| 1. If yes, to either question 12 or question 13, was your highest qualification… | A degree level or above / Or another kind of qualification? | |

**Supplementary Data S2**

Patient interview guide

| Part 1. Current monitoring strategy |
| --- |
| Can you tell me about when you were first advised to have these blood tests?  How did you feel about initially having blood tests very frequently?  How was your experience of the process of having frequent blood-tests? |
| Once you reached the correct dose for your medication, the frequency of your blood tests was reduced. How do you find having these blood tests every [frequency]?  Explore concerns / benefits of this frequency |
| On the questionnaire you said you had [never missed / missed a blood test x times in the past 12 months]. What led you to attend these blood tests regularly / what were the reasons for you missing some blood tests?  Explore experience attending blood tests; any difficulties. |
| On a scale of 1-10 (1 = not at all; 10 = extremely), how important is it for you to continue with these blood tests as frequently as you currently do?  Explore reasons for number; why not higher/lower; what is it that is/isn’t important |
| How would you feel about the frequency of your blood tests being tailored to your personal risk of experiencing a problem caused by these medications? |
| What would be important for you to know if your GP or hospital doctor suggested changing the frequency of blood-tests according to your personal risk of experiencing such a problem? |
| Part 2. Proposed monitoring strategy |
| We are now going to move on to talk about the proposed changes to how we monitor people on immune suppressing medications.  Currently, once the correct dose of an immune suppressing medication is reached for a patient, it is recommended they continue with regular blood tests. Most people are recommended to have a blood test every three months, regardless of their personal risk of experiencing a problem from the immune suppressing medication they take. Someone with a low risk of getting blood, liver or kidney damage is offered a blood test as often as someone who is at a higher risk. We know that although it is important to continue to check for problems in the long-term, they are not common after six months of treatment.  Our research team have made a new plan to monitor people so that it will still detect problems but is more cost-effective. They have looked at what things may increase the chance of someone experiencing blood, liver, or kidney damage from these medications, and have used this information to create a calculator that determines how often a blood test is needed based upon each person’s own risk of experiencing such problems. At present, this is only being used for research purposes but if it is adopted for use in the NHS then for many people, this would mean they don’t need a blood test as often as three-monthly, while some will need a blood test more often.  We are proposing that this is how we should monitor people on immune suppressing medication in the future.  [For conventional DMARDs only] I’m now going to ask you a few questions, the answers to which will tell me how often the calculator determines it is safe for you to have a blood test based on your own risk of experiencing a side-effect from your medication. We will then discuss your views on this. If 100 people like you were given [medication] for a one-year period, [x] person(s) would need to stop their treatment due to abnormal blood test results.  *Complete risk calculator for patients taking conventional DMARDs*  The research so far suggests that it would be best use of NHS resources for you to receive a blood test either six-monthly, once a year or once every two years. However, if there is a longer gap between the tests, potential side-effects may be detected quite late so it’s important for us to understand the gap in testing that would be acceptable to you. |
| Considering this information, how do you feel about having a blood test [frequency] going forward?  Explore each potential frequency; concerns / benefits about reducing frequency |
| Overall, how do you feel about the new plan to monitor people on immune suppressing medications compared to the current way?  Explore views / concerns on moving straight to accepted frequencies |
| Again, what would be important to know if your doctor suggested changing?  How would you want that to be communicated to you? |

**Supplementary Data S3**

Healthcare professional interview guide

| Part 1. Current monitoring strategy |
| --- |
| Can you tell me about your involvement in the current monitoring of patients with inflammatory conditions?  Initial monitoring / long-term monitoring; which DMARDs |
| For the next few questions, I’d like you to consider the long-term monitoring plan for these patients, after the first 6-9 months of treatment, once they are on a stable dose. What are your views on the frequency that these monitoring blood tests are offered? |
| What difficulties, if any, have you encountered in providing blood tests this frequently to patients?  Are there any practical challenges or difficulties in implementing these tests? |
| Are there any specific risks you feel, in providing blood tests this frequently?  Explore how risks are managed in practice |
| What are your views on how well the tests pick up true positive results (i.e. correctly identifies where a marker is out of range) and true negative results (i.e. correctly identifies where a marker is in range)?  If they feel there are lots of false positives or false negatives – explore impact of this |
| Are there any specific benefits in providing blood tests this frequently? |
| Part 2. Proposed monitoring strategy |
| We are now going to move on to discuss a new proposed monitoring strategy.  Currently, once the correct medication dose is reached, all patients, regardless of their individual risk of a side-effect, are recommended and invited to have a blood test on a regular basis, typically every three months. We know that although it is important to continue monitoring side effects in the long-term, they are rarely experienced after six months of starting these medicines and it is costly for the NHS to provide blood tests to all patients at this frequency.  We are looking to create a new monitoring strategy that is cost-effective but still safe for patients. Prior to these interviews, our research team looked at the various risk factors for experiencing a side effect and calculated how often patients would need a blood test based upon these risks. As part of this research, the team also created a risk-score calculator. The calculator generates a personalised risk-score, telling us the likelihood that someone will have to stop their treatment due to an abnormal test result. This is based on the type of immune-suppressing medication they take and their answers to the factors that increase and decrease the risk of an abnormal result.  *Show risk-calculator on screen and go through the factors that make up the score.*  I’m now going to show you some vignettes which describe a few patients, their risk-scores and the frequencies they could receive a blood test. I’d like you to read them and we will then discuss your views on the new strategy. It is important we understand how you feel about this change, as we want to make a recommendation that is acceptable to healthcare professionals.  The risk score reflects a person’s overall risk for experiencing a side effect from an immunosuppressant, taking into account all risk factors. For the next questions, I’d like you to focus on the risk-score given for each patient. Risk factors on their own are poor predictors of someone’s risk of side effects, but when combined they work well to predict risk so it’s important you consider the score over individual risk factors. |
| Based on this vignette, we’d like to know what frequency would be acceptable to you as a clinician.  Explore concerns / benefits of implementing each frequency in practice |
| Overall, how do you feel about the new monitoring strategy compared to the current strategy?  What do the risk scores mean to you?  Explore what seems high risk/low risk. |
| If this new monitoring strategy was adopted by national guidance, how would you like the changes to clinical practice communicated to you?  Explore what would be important to know about it to help them / patients |

**Supplementary Data S4**

Examples of patient scenarios shown to healthcare professionals


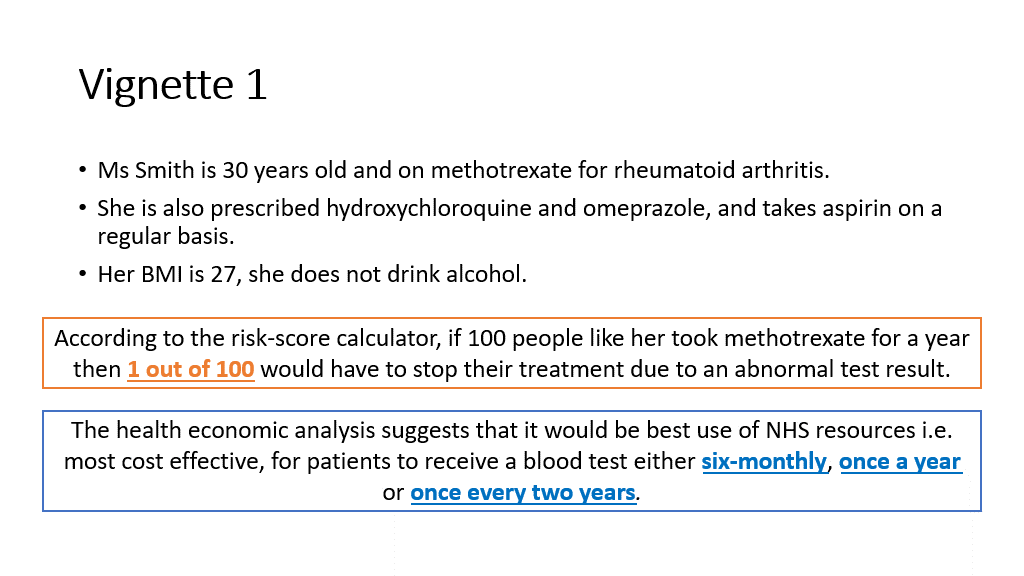


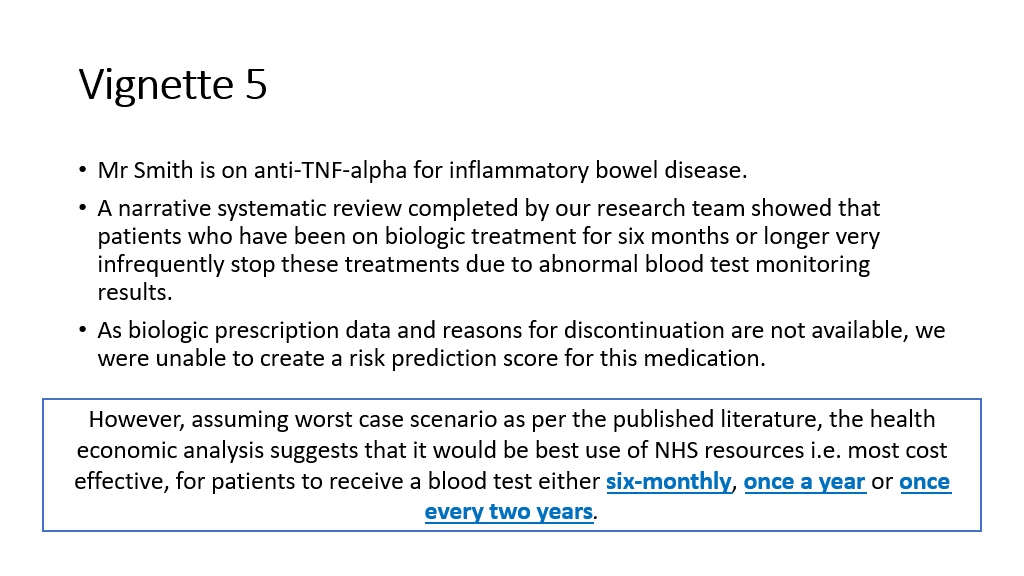

Supplement: keae175_Supplementary_Data [file keae175_supplementary_data.docx]
